# Homœopathy

**Published:** 1856-09

**Authors:** 


*Homœopathy.*

Men are familiar with the advertisements of disguised medicines which continually appear, and sink into neglect, again to be brought forward under a new name, and perhaps slightly altered in appearance. It is now difficult to find a person who credits what is urged in favor of any one of these nostrums; and they are used, for the most part, when a cheap article is made choice of, such directions as go with them being cheaper than the advice of a physician.

They manage such things differently in Germany. Besides the usual extravagant pretensions, each one is there especially anxious to show that his own nostrum is in the height of fashion, and is used by the nobility everywhere. They endeavor to get the recommendation of some prince or baron, and sometimes with success. The same course was followed by those charlatans who got up the system of medical practice called homœopathy; and when it was imported into this country, that course being continued here, some of the American "aristocracy" were a little, a very little, imposed on thereby. But if any wish to inquire, they can find that homœopathy has for sixty years been scouted by nearly all persons of the higher ranks in Europe; and that the only evidence that it ever was fashionable, is derived from some homœopathic source. The kind of evidence that a lawyer delights in, is that which is wrung from the witnesses of his opponent; and we in this paper rely chiefly on the writings of the homœopathists themselves. Their periodicals are all in reality quack advertisements, under the disguise of scientific publications, as any well-informed person may ascertain by examination. Though no more credible than such advertisements in other cases, especially when they speak of their success, yet that which they contain adverse to their own pretensions is not unworthy of observation. They continually complain that they are subjected to the laws against quackery, which exist in most European countries, and are enforced more or less strictly in different places.

From the *British Journal of Homœopathy*, published at London, Oct.,

1853, p. 665, it appears that no homœopathist ever practised in the large republican city of Frankfort before 1848; that one who began business there, about that time, was soon expelled from the city by the magistrates, solely on account of his method of practice; that he then took up his residence in a neighboring village, within the bounds of Hesse Cassel, and visited his patients in the city as before; and that for these visits he was fined and compelled to leave the neighborhood. From the silence of subsequent publications it would seem that no homœopathist has been permitted to practise there since.

In the same quarterly, for July, 1853, p. 485, it is said that homœopathy was never practised in Rome until Dr. Wahle removed there in 1843, after his residence at Leipsic had been made disagreeable by "persecution;" and that for a long time he met with great difficulties in getting permission to begin business, though latterly his practice was large. It appears from the article that the time when those difficulties were removed, and his practice became large, was at the setting up of the last Roman Republic in 1848. At the restoration of the Pontifical government, his practice seems again to have been interfered with, for it is mentioned that he had leisure to travel into the north and Germany.

In the *North American Homœopathic Journal*, published in New York, May, 1852, p. 127, speaking of late political events, it is said that in France, Italy and Germany, revolutions and still more reactions, have hindered the progress of homœopathy; and that tyrants, at the instigation of their court physicians, will not foster it. From this it should perhaps be inferred, that after the revolutionary movements of 1848 were subdued, the homœopathists were again subjected to the degradation from which the outbreak had relieved them, and that the laws against them were executed more rigorously than before, because some had neglected homœopathy to meddle with state affairs. In the *Quarterly Homœopathic Journal*, published at Boston, July, 1850, p. 300, it is said that Dr. Wurmb, one of the first homœopathists in Vienna, was a captain in the students' legion, during the revolution, and took part in one of the battles; and that the reaction has nearly destroyed him.

The *Philadelphia Journal of Homœopathy*, for Feb., 1854, p. 688, says that homœopathic practice, after a comparatively free course in Bavaria for twenty years, was interdicted in 1842 in all the public institutions of the kingdom; and that in 1848, the year of the revolution, the prohibition was removed, but was again imposed soon afterwards.

The *British Journal of Homœopathy*, for April, 1855, p. 328, says that homœopathy was prohibited in Austria in 1819; and though that prohibition was said to have been removed a long time afterwards, their journals also say other things inconsistent with that assertion. The *Quarterly Homœopathic Journal*, for July, 1850, p. 305, speaking of Austrian tyranny since the revolution, says, "the rescript of the government, permitting the homœopathic practice to all physicians, has never been allowed to be published." That rescript would seem to have been the product of the revolution, and to have lost its force when the emperor recovered his power; and, it is said, on the same page, that some homœopathists, "in order not to be discovered, pretend to prescribe allopathic medicines which their patients never take, using homœopathic medicines all the time according to the actual directions." Instead of appearing openly, as men of fashion like to appear, they are obliged to hide their doings from the police. Still, it has repeatedly been written that homœopathy is more flourishing in Austria than on any other part of the European continent; and since they are reduced to such expedients, how must they manage in places where they are yet more restricted? As astrologers and other fortune tellers have managed to lurk in the shade when their arts are prohibited, so homœopathy has by some means prolonged a degraded existence without being able to convince the magistrates that its pretensions are true. Were they true, it must have been eagerly received by all, more than fifty years ago. It is now practised by only a few hundred in all Europe, while of regular physicians there are hundreds of thousands.

The *North American Homœopathic Journal* for May, 1853, p. 271, contains an extract from the *Zeitschrift für Homœopatische Klinik*, published in Germany. It gives an account of the state of homœopathy in 1852. It recognizes its low state in Prussia, and other northern parts of Germany; also in France, Sweden, Denmark and Russia, but says it flourishes in Austria, Bavaria and other southern countries of Germany. It says that in England it flourishes more than in Germany itself; that the number of practitioners is there large, and that, "above all, America seems to have given it the warmest embrace." The *British Journal of Homœopathy* for July, 1853, p. 480, speaking of a register of the names of their practitioners in Europe and America, says "the latter, especially, is a most formidable list." We have here important data. The United States, it would seem, contain more homœopathic practitioners than Europe, England more than Germany, and the south of Germany more than the north.

The *North American Journal of Homœopathy* for Nov., 1852, p. 493, contains a list of their practitioners in New York and the principal Atlantic cities. The editors show an anxiety to make the number appear as large as possible. The number given for New York city is 62; for the rest of the State, 242; Philadelphia, 53; Boston, 20; Providence, 9; Baltimore, 10; Washington, 2. Total, 398. This is the list so much admired by the Europeans; and as in the scattered population of the south, and of the country towns of the west and the east, they fail in finding adherents sufficient for support, there is no reason to suppose that one hundred others could have been reckoned in this country.

The *Quarterly Homœopathic Journal* for April, 1850, p. 285, quoting the *British Journal of Homœopathy* for the January preceding, gives a list of the British practitioners. The number is 48 in London, 51 in the rest of England, 10 in Scotland, and 7 in Ireland. Total, 116. Though the number in Germany they thus show to be insignificant, no credible evidence is found that it ever was larger. The Central Homœopathic Union of Germany, which collects to its meetings as many as possible of the practitioners of that country, held its annual meeting in 1854 at Weimar, in a densely populous region traversed by railroads. The nmber present was published, for it seems to have been unusually large. It was twenty-seven, of whom two were styled apothecaries, and one a veterinary surgeon. The year previous, the meeting was appointed at Hesse Cassel; but the homœopathists were prohibited by the sovereign of that country from assembling within his dominions.) See *Brit. Jour. Hom.*, Oct., 1853, p. 668; and Oct., 1854, p. 683.

As men of eminence have in some instances resorted to homœopathy, in times of doubt and terror, so they have resorted to dealers in false pretensions of other kinds. The last-named journal for Oct., 1853, p. 670, publishes a certificate alleged to have been signed by the Marshal de St. Arnaud, declaring that he had just been completely cured, by homœopathy, of a disease that for fifteen years had seriously troubled him. The disease referred to must have been the one which soon afterwards proved fatal to him, at the close of the battle of Alma. The same page contains his answer to a request that he would use his influence to relieve the homœopathists from the legal disgrace that burdens them in France; but though he was then Minister of War to Louis Napoleon, he with some ambiguous, courtly expressions of regard, declined to take the first step in the matter. Though he consulted them as he might have consulted fortune tellers, he seems to have held them in no higher estimation.

The degree of success, which has attended them in this country, has resulted chiefly from their groundless assertions that they are everywhere patronized by the most fashionable, the most intelligent, and the most learned; and in fact some of the American literati appear to have been rather easily captivated by their elegantly printed publications, and even half convinced by their external appearance alone. There is a sort of natural philosophy, admired by many, called transcendentalism, whether correct or otherwise it is not to the purpose here to inquire; but the chief of the works on homœopathy are written in the style of transcendentalism, and contain many of its peculiar expressions and modes of thought. Yet it is not transcendentalism, but an imitation of it merely; so, of course, the German metaphysicians consider it, or they would not have despised it, as they all do, except some few of very peculiar acuteness of penetration. Had they received it, the practice of it would never have been forbidden. It is not only counterfeit science, and counterfeit fashion, but counterfeit transcendentalism. The American votaries of this philosophy have, some of them, been prone to take the counterfeit for genuine; and it is not rationally to be expected that such as have resided not many weeks in the neighborhood of German libraries, and are but moderately well versed in the things of Germany, can have developed those powers of detecting German counterfeits, which the natives of the land themselves possess.—*Boston Medical and Surgical Journal.*

